# Nociplastic Features Associate with Poorer Function in Aging Veterans with Chronic Low Back Pain

**DOI:** 10.1093/geroni/igaf122.2030

**Published:** 2025-12-31

**Authors:** Victoria Powell, Jinkyung Kang, Andrzej Galecki, Daniel Clauw, Pooja Lagisetty, Maria Silveira, Caroline Logue, Sarah Krein

**Affiliations:** University of Michigan, Ann Arbor, Michigan, United States; University of Michigan, Ann Arbor, Michigan, United States; University of Michigan, Ann Arbor, Michigan, United States; University of Michigan, Ann Arbor, Michigan, United States; University of Michigan, Ann Arbor, Michigan, United States; University of Michigan, Ann Arbor, Michigan, United States; LTC Charles S. Kettles VA Medical Center, Ann Arbor, Michigan, United States; University of Michigan, Ann Arbor, Michigan, United States

## Abstract

Chronic low back pain (CLBP) is a common cause of disability in aging veterans. CLBP is increasingly recognized as one of several overlapping pain conditions where nociplastic pain is likely the predominate mechanism underlying pain and associated disability. Nociplastic features include pain that is chronic, widespread, and accompanied by additional symptoms such as fatigue, sleep disorders, and mood disturbance. However, to what extent clinically significant nociplastic features interfere with function in aging veterans is unknown. We conducted a nationally representative cross-sectional survey of veterans 50-89 years old with ≥2 outpatient encounters for low back pain. The Veterans RAND 12-Item Health Survey was used to measure general functioning. The Fibromyalgia Survey Questionnaire was used to quantify nociplastic features. Of n = 800 invited veterans, 45.1% returned surveys. Nociplastic features were common, with over one-third (35.8%) reporting nociplastic features severe enough to screen positive for fibromyalgia. More severe nociplastic features were significantly associated with poorer physical (β =-0.30 (-0.49, -0.12), p < 0.0001) and psychological functioning (β=-0.639 (-0.93, -0.35), p < 0.0001) after adjusting for demographics, comorbidities, pain intensity, and opioid use. Our findings support that in aging Veterans with CLBP, nociplastic features are commonly present, and in one-third of cases severe enough to meet criteria for fibromyalgia, the prototypical nociplastic pain disorder. Reporting more nociplastic features was independently associated with worse physical and psychological function in a dose-dependent manner. Future studies, including trials of new pain interventions aimed at improving or maintaining functional status, should examine whether the severity of nociplastic features predicts treatment response.

